# Incidence and predictors of annual chlamydia testing among 15–29 year olds attending Aboriginal primary health care services in New South Wales, Australia

**DOI:** 10.1186/s12913-015-1116-5

**Published:** 2015-09-30

**Authors:** Simon Graham, Rebecca J. Guy, James S. Ward, John Kaldor, Basil Donovan, Janet Knox, Debbie McCowen, Patricia Bullen, Julie Booker, Chris O’Brien, Kristine Garrett, Handan C. Wand

**Affiliations:** Kirby Institute, UNSW Australia, Sydney, NSW 2052 Australia; Centre for Epidemiology and Biostatistics, School of Population and Global Health, University of Melbourne, Melbourne, VIC 3052 Australia; South Australian Health and Medical Research Institute, Adelaide, SA 5000 Australia; Sydney Sexual Health Centre, Sydney Hospital, Sydney, NSW 2000 Australia; Aboriginal Community Controlled Health Service, New South Wales, NSW Australia

**Keywords:** Indigenous, STI, Sexual health, Quality improvement shimmer

## Abstract

**Background:**

For the past two decades, chlamydia has been the most commonly notified infectious disease among young people (15–29 year olds) in Australia, the United States of America and the United Kingdom and rates have increased annually in these three countries. In Australia, rates of chlamydia are three times higher in Aboriginal compared with non-Aboriginal people. Australian sexually transmissible infection guidelines recommend annual chlamydia testing for 15–29 year old females and males. This analysis will examine the incidence and predictors of annual chlamydia testing in 15–29 year olds attending four Aboriginal Community Controlled Health Services (ACCHS) in Australia.

**Methods:**

From 2009–2011, attendance and chlamydia testing data were extracted from the patient system to calculate the number and proportion of 15–29 year olds that were tested for chlamydia and that tested positive for chlamydia by gender (male, female), age-group (15–19, 20–24, 25–29 years), Aboriginal status (Aboriginal, non-Aboriginal people) and by the four ACCHSs sites (1, 2, 3 and 4). A cohort was created to calculate the incidence rate per 100 person-years (PY) and predictors of an annual chlamydia test (a test within 12-months of a previous test/visit) by the above factors using Cox regression. Unadjusted and adjusted hazard ratios (AHR) and their 95 % confidence intervals (CIs) and p-values were calculated with significance at *p* < 0.05.

**Results:**

From 2009–2011, there were 2896 individuals who attended the four ACCHSs. Overall , 17 % (22 % of females and 10 % of males) were tested for chlamydia and 9 % tested positive (8 % of females and 14 % of males). The median time to an annual chlamydia test was 10.7 months. The cohort included 2318 individuals. Overall the incidence rate of an annual chlamydia test was 9.1 per 100 PY (11.6 in females and 5.8 in males). Predictors of an annual chlamydia test were being female (AHR: 1.7, 95 % CI: 1.2-2.2, *p* < 0.01), being 15–19 years old (AHR: 1.6, 95 % CI: 1.1-2.3, *p* < 0.01) and attending ACCHS site 2 (AHR: 3.8, 95 % CI: 1.8-8.0, *p* < 0.01).

**Conclusions:**

This analysis highlights that opportunistic STI testing strategies are needed to increase annual chlamydia testing in young people; especially males.

## Background

Chlamydia is a sexually transmissible infection (STI) and for the past two decades it has been the most commonly notified infectious disease among young people in Australia [1], the United States of America (US) [2], the United Kingdom (UK) [3] and New Zealand [4]. In Australia, chlamydia notification rates are higher in females, 15–29 year olds and those living in regional and remote areas [1]. Chlamydia notification rates are three times higher among Aboriginal and Torres Strait Islanders (hereafter ‘Aboriginal’) compared with non-Aboriginal people in Australia and rates are increasing [1, 5]. Higher rates of chlamydia have also been reported among Indigenous compared with non-Indigenous people in Canada, the US and New Zealand [4, 6, 7]. Reasons for these higher rates could be attributed to lower access to primary health care services, and cultural and social determinants of health [8].

An estimated 85 % of chlamydia infections are asymptomatic and as a result regular STI testing is an important prevention strategy to detect and treat infection early, reduce poor health outcomes from developing and to prevent transmission to others [9]. Untreated chlamydia infection can lead to pelvic inflammatory disease and infertility [10, 11]. STI guidelines in the US [2] and the UK [3] recommend annual chlamydia testing of 16–24 year old females; however Australian STI guidelines recommend annual chlamydia testing of 15–29 year old females and males [12].

To decrease the prevalence of chlamydia, modelling studies in Australia and the UK have suggested that an annual chlamydia testing rate of 30 % or greater among 15–24 year old females is needed [13, 14]. However, a modelling study conducted in remote Aboriginal communities in Australia, where chlamydia is endemic suggested an annual chlamydia testing rate of 60 % or greater would be required to reduce the prevalence of chlamydia by 9 % [15].

Primary health care centres are recognised as playing an important role in STI testing, treatment and management [16, 17]. In Australia, Aboriginal Community Controlled Health Services (ACCHS) provide culturally appropriate primary health care to Aboriginal people with an estimated 142 ACCHSs across Australia and 42 in the state of New South Wales (NSW) [18]. A community-based survey of 16–30 year old Aboriginal people in NSW found that greater than 50 % of participants preferred to receive STI testing, treatment and education at an ACCHSs compared with non-Aboriginal primary health care services and sexual health clinics [17]. This highlights that ACCHS are the preferred health care provider for young Aboriginal people in NSW.

A cross-sectional study of 16–29 year olds attending non-Aboriginal primary health care centres in Australia found that 8 % were tested for chlamydia, with higher testing rates in females than males (12 % vs 4 %, *p* < 0.01) [16]. However this cross-sectional study cannot determine if the same people are tested every year or if different people are being tested. To more accurately examine annual chlamydia testing of the same person, we used a longitudinal study design to estimate the incidence and predictors of an annual chlamydia test among 15–29 year olds who attended four ACCHSs in NSW Australia.

## Methods

### The aboriginal population

In 2014, the Australian Institute of Health and Welfare (AIHW) estimated that there were 713,600 Aboriginal people in Australia which accounted for 3 % of the Australian population [8]. The number of Aboriginal people varied across the Australian jurisdictions with the largest estimated population residing in NSW (220,902) [8]. Nationally, the median age of the Aboriginal population is 21 years compared with 37 years for the non-Aboriginal population [8]. Aboriginal compared with non-Aboriginal people have higher rates of chronic and other communicable diseases, higher rates of unemployment; lower levels of home ownership, school completion and life expectancy [8]. To reduce the disparity between Aboriginal and non-Aboriginal people the Australian government has released a number of national strategies including the *Fourth National Aboriginal and Torres Strait Islander Blood-Borne Viruses (BBV) and Sexually Transmissible Infections (STI) Strategy, 2014–2017* [19].

This study was conducted prior to the implementation of a sexual health quality improvement (QIP) project commencing in four ACCHSs in NSW known as SHIMMER [20]. SHIMMER aimed to increase STI (chlamydia, gonorrhoea, syphilis, HIV) and hepatitis (hepatitis B and C) testing and improve the management of these infections. To assist in achieving the aims of SHIMMER, this longitudinal analysis was conducted to assist in developing STI testing and management strategies. The QIP used in SHIMMER commenced in March 2012. This analysis focusses on chlamydia because it has the highest prevalence among young Aboriginal people and because we wanted to use chlamydia as a starting point to develop the STI testing and management strategies and then slowly add other testing strategies that would address the other infections.

SHIMMER was conducted in collaboration with four ACCHSs in NSW to ensure Aboriginal people were included in the decision making process, maintained ownership of their data and were integral to the implementation of the SHIMMER project. The four ACCHSs varied in size and scope of medical, allied health and health education services provided to local Aboriginal communities. They were all located in regional areas of NSW and serviced different Aboriginal communities. The four ACCHSs were located large distances from each other to limit the possibility of young people attending multiple ACCHSs. They also had independent management and protocols for medical and allied health services provided to the local Aboriginal communities.

### Data extraction

A data extraction tool called the ‘GeneRic Health Network Information Technology for the Enterprise’ (GRHANITE™) [21] was installed onto the electronic patient system at the four ACCHSs. GRHANITE™ extracted de-identified patient attendance and STI testing data through the internet. Only medical consultations with a doctor or nurse were included, with allied health consultations such as dental and physiotherapy excluded.

### Statistical analysis

Data from 2009–2011 were used to calculate the following among 15–29 year olds:

1. The number and proportion of individuals that attended (**attendance**) by the following factors:
  - Gender (female, male)
  - Age-group (15–19, 20–24, 25–29 years)
  - Aboriginal status (Aboriginal, non-Aboriginal)
  - ACCHS site (1,2,3 and 4)
2. The number and proportion of individuals who were tested for chlamydia by the above mentioned factors (repeat visits and tests of the same person were remove - **proportion tested overall**).
3. The number and proportion of individuals who tested positive overall by the above mentioned factors (repeat positives and tests of the same person were removed -**positivity**).
4. The proportion of individuals who were tested at least once for chlamydia in each year (in 2009 and in 2010 and in 2011) by gender (repeat tests and visits of the same person within a 12-month period were removed) - **proportion tested annually**).

We used a chi-squared test with statistical significance at *p* < 0.05 to assess if there were any differences in chlamydia testing in females and males by the above mentioned factors

**Table 1:** Number and proportion tested for chlamydia and positivity in 15–29 year olds, 2009-2011

Demographic factors and ACCHS site	Individuals	Tested for chlamydia	*P*-value*	Tested positive
	n (%)	n (%)		n (%)
Overall	2896 (100)	495 (17)		47 (9)
		Males		
	1223 (42)	119 (10)	<0.01	17 (14)
Age-group (years)				
15-19	481 (39)	56 (12)	0.14	9 (16)
20-24	354 (29)	33 (9)		4 (12)
25-29	388 (32)	30 (8)		4 (13)
Aboriginal status				
Aboriginal	1023 (84)	110 (11)	<0.01	15 (14)
Non-Aboriginal	200 (16)	9 (5)		2 (22)
ACCHS site				
1	220 (18)	5 (2)	<0.01	0 (0)
2	178 (15)	27 (15)		4 (15)
3	514 (42)	77 (15)		12 (15)
4	311 (25)	10 (3)		1 (10)
		Females		
	1673 (58)	376 (22)		30 (8)
Age-group (years)				
15-19	605 (36)	130 (21)	0.07	20 (15)
20-24	578 (35)	148 (26)		8 (5)
25-29	490 (29)	98 (20)		2 (2)
Aboriginal status				
Aboriginal	1323 (79)	316 (24)	<0.01	27 (9)
Non-Aboriginal	349 (21)	60 (17)		3 (5)
ACCHS site				
1	402 (24)	16 (4)	<0.01	3 (18)
2	204 (12)	42 (20)		5 (12)
3	673 (40)	249 (37)		17 (7)
4	394 (24)	69 (17)		5 (7)

*p-value with significance at *p* < 0.05

We then created a cohort to assess the incidence rate of an annual chlamydia test and predictors of an annual chlamydia test from 2009–2011. An annual chlamydia test was defined as a test within 12-months of a previous test/visit. Those aged 15–29 years entered the cohort at their first chlamydia test in 2009 or if not tested in 2009, then those who attended from 1st January 2010. We selected this date because national STI guidelines in Australia recommend annual chlamydia testing of 15–29 year olds [12] and to ensure twelve-months had passed since their previous chlamydia test. If a person was 29 years old when they entered the cohort in 2009 or 2010, they stayed in the cohort until they were tested even if they turned 30 year olds.

Using this cohort we calculated the following among 15–29 year olds:

1. The incidence rate of annual chlamydia testing per 100 person-years (PY) overall and by the above mentioned factors and by Year (in 2010 and in 2011 – **incidence rate of annual chlamydia testing**)
2. The predictors of annual chlamydia testing by the above mentioned factors – **predictors of annual chlamydia testing**)

To examine the predictors of annual chlamydia testing we used univariate and multivariate Cox regression. The regression analysis was restricted to the variables that were collected by GRHANITE^TM^. Unadjusted and adjusted hazard ratios (HR) and their 95 % confidence intervals (CIs) and p-values were calculated with statistical significance at *p* < 0.05.

Data were analysed using STATA 12 statistical software (STATA Corporation, College Station TX).

### Ethical approval

The SHIMMER project received ethical approval from the Aboriginal Health & Medical Research Council of NSW and the University of New South Wales Human Research Ethics Committee. We also received approval from each participating ACCHSs Boards and signed agreements with each ACCHSs detailing issues such as confidentiality, privacy, consent, use of patient data, publications, and roles and responsibilities. The SHIMMER project was funded by the NSW Ministry of Health.

## Results

### Attendance

From 2009–2011, 2896 individuals aged 15–29 years attended the four ACCHSs. A higher proportion were aged 15–19 years (38 %), Aboriginal (81 %) and attended ACCHSs site 3 (41 %, Table 1).

### Proportion tested overall

Overall, 17 % of individuals were tested for chlamydia, 22 % of females and 10 % of males (*p* < 0.01, Table 1). Among males, a higher proportion of 15–19 year olds (12 %) were tested for chlamydia compared with the other two age-groups; however this was not statistically significant (*p* = 0.14). A higher proportion of Aboriginal compared with non-Aboriginal males were tested (11 % vs 5 %, *p* < 0.01). Among females, a higher proportion of 20–24 year olds (26 %) were tested for chlamydia compared with the other two age-groups (Table 1); however this was not statistically significant (*p* = 0.07) and a greater proportion of Aboriginal females compared with non-Aboriginal females were tested (24 % vs 17 %, *p* < 0.01).

### Proportion tested annually

The proportion of individuals tested for chlamydia in each year of the study was similar; from 7 % in 2009 to 6 % in 2010 and 8 % in 2011. Among females, 9 % were tested in 2009, 10 % in 2010 and 10 % in 2011and among males, 3 % were tested in 2009, 2 % in 2010 and 7 % in 2011.

### Incidence rate of an annual chlamydia test

**Fig. 1:**
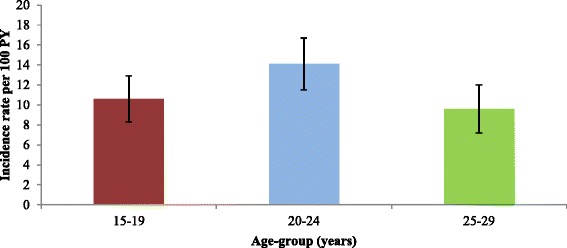
Incidence rate and 95 % confidence intervals of chlamydia testing in females by age-group, 2009-2011

**Fig. 2:**
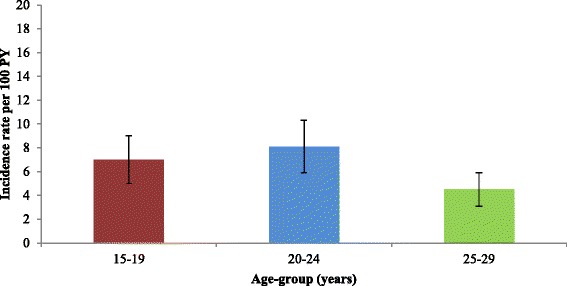
Incidence rate and 95 % confidence intervals of chlamydia testing in males by age-group, 2009-2011

A total of 2318 individuals were included in the cohort and the median time from entering the cohort to a chlamydia test was 10.7 months. Overall, the incidence rate of an annual chlamydia test per 100PY was 9.1; higher in females, 20–24 year olds, Aboriginal people and those who attended ACCHSs site 3 (Table 2). Among females and males, the highest rate of an annual chlamydia test was in 20–24 year olds (Figs. 1 and 2).

**Table 2:** Incidence rates and predictors of chlamydia testing in 15–29 year olds, 2009-2011

Demographic factors, year and ACCHS site	Incidence rate^a^ (95 % CI)^b^	Unadjusted hazard ratio (95 % CI)^b^	*p*-value*	Adjusted hazard ratio (95 % CI)^b^	*p*-value*
2009-2011	9.1 (8.1-10.3)				
Year					
2010	9.6 (7.8-11.8)				
2011	8.9 (7.7-10.3)				
Gender					
Female	11.6 (10.1-13.3)	1.5 (1.1-1.9)	<0.01	1.7 (1.2-2.2)	<0.01
Male	5.8 (4.6-7.3)	1	-	1	-
Age-group (years)					
15-19	9.0 (7.4-11.0)	1.3 (0.9-1.8)	0.06	1.6 (1.1-2.3)	<0.01
20-24	11.0 (9.1-13.2)	1.2 (0.9-1.6)	0.24	1.3 (0.9-1.8)	0.11
25-29	7.1 (5.6-9.1)	1		1	
Aboriginal status					
Aboriginal	9.7 (8.6-11.1)	1.1 (0.8-1.6)	0.50	1.2 (0.9-1.8)	0.05
Non-Aboriginal	6.3 (4.5-8.8)	1	-	1	-
ACCHS site					
1	1.1 (0.5-2.2)	1.6 (0.7-3.5)	0.25	1.6 (0.7-3.6)	0.22
2	13.6 (10.4-17.8)	3.4 (1.6-7.1)	0.01	3.8 (1.8-8.0)	<0.01
3	14.0 (12.0-16.4)	2.0 (0.9-4.4)	0.07	2.2 (0.9-4.7)	0.05
4	7.6 (5.9-9.8)	1		1	

^a^Incidence rate per 100 person-years, ^b^Hazard ratio and 95 % confidence interval, *p-value – significance at *p* < 0.05

### Predictors of annual chlamydia testing

Independent predictors of an annual chlamydia test were being female, being 15–19 year olds, and attending ACCHS site 2 (Table 2).

### Positivity

Over the three-year period, there were 495 individual chlamydia tests with 9 % testing positive (Table 1). Although a higher number of positive chlamydia tests were in females than males (30 vs 17), a higher proportion of males than females tested positive (14 % vs 8 %, Table 1). Among males, the highest positivity was in 15-19 year olds (16 %) compared with the other two age-groups and in females, positivity was highest in 15–19 year olds (15 %, Table 1).

## Discussion

This study found that 17 % of young people were tested for chlamydia over a three-year period and 9 % had an annual chlamydia test. Although a higher number of females than males tested positive for chlamydia a higher proportion of males than females tested positive. Independent predictors of an annual chlamydia test were being female, being 15–19 years old and attending ACCHS site 2.

A strength of this study was that it examined chlamydia testing by using routinely collected attendance and STI testing data from the electronic patient system at four ACCHSs which provided a large sample size for the analysis. There are a number of limitations of our study including; attendance and chlamydia testing data that were not entered into the electronic patient record system were excluded. It is possible that some individuals may have undergone repeat testing at non-Aboriginal primary health care centres; therefore we may have under-estimated annual testing rates. The regression analysis did not include the testing of other STIs which could have been a factor in predicting chlamydia testing. GRHANITE^TM^ could not extract the reason why young people attended the ACCHS and this could have influenced the predictors of chlamydia testing. We were not able to identify those individuals who were residents of the local Aboriginal communities compared with visitors to the Aboriginal communities; which could result in our study under-estimating annual chlamydia testing rates as local residents are able to return annually to be tested compared to visitors to the communities.

In our study, the incidence rate of an annual chlamydia test in females was 11.6 per 100PY. Two other longitudinal studies have examined the incidence rate of an annual chlamydia test in females; one in the US and another in Australia [22, 23]. The US study found that the incidence rate of an annual chlamydia test ranged from 2.1 per 100 woman-years in 15–19 year old females to 13.5 per 100 woman-years in 25 year old females [22] and the Australian study found the incidence rate of an annual chlamydia test was 4.4 per 100 woman-years in 16–29 year old females attending primary health care centres and sexual health clinics [23]. Overall, the annual testing rate in our study was higher compared with the US and other Australian study.

Being female was an independent predictor of having an annual chlamydia test in our study. Higher chlamydia testing rates in females compared with males has been a common finding in a number of other studies [16, 24]. One factor influencing this could be the types of consultations that females are having, such as reproductive health consultations (contraception, Pap smear, pregnancy). A study conducted in primary health care centres in Australia found that reproductive health consultations were highly associated with chlamydia testing [16]. To increase chlamydia testing in males the ACCHS could include chlamydia testing in adult health checks (AHCs) if not already included. AHC are a general health assessment offered in ACCHS [25]. For every completed AHC the ACCHS can receive $212 from the Australian government’s Medicare Benefits Scheme. A completed AHC can be charged every nine-months [25]. A clinical audit was undertaken in the SHIMMER project which highlighted that 50 % of AHCs among young males included a chlamydia test; however only 16 % of the males were offered an AHC [26]. Offering more AHC could be an effective STI testing strategy to increase chlamydia testing in young males and among females who are not attending for reproductive health reasons.

In our study, 9 % of 15–29 year olds had an annual chlamydia test. The importance of regular chlamydia testing is highlighted by three factors; that chlamydia re-infection is common (~22 %) [27], that 85 % of chlamydia infections are asymptomatic [9] and that among those females who had untreated chlamydia infection, 10 % developed pelvic inflammatory disease with 12-months [28]. As chlamydia re-infection is common annual chlamydia testing is an important prevention strategy with Australian STI guidelines recommending a re-test for chlamydia three-months after being diagnosed [12]. These factors highlight the importance of developing STI testing strategies for young people that will identify new infections early and provide treatment and education.

## Conclusions

This analysis highlights that opportunistic STI testing strategies could be used by the ACCHSs to increase annual chlamydia testing in young attendees; especially males.

## Abbreviations

Aboriginal: Aboriginal and Torres Strait Islander
ACCHS: Aboriginal community controlled health services
AIHW: Australian Institute of Health and Welfare
AHC: Adult health check
BBV: Blood borne viruses
CIs: 95 % confidence intervals
GRHANITE^TM^: Generic Health Network Information Technology for the Enterprise
HR: Hazard ratio
NSW: New South Wales
PY: Person-years
QIP: Quality improvement program
STI: Sexually transmissible infection
SHIMMER: Sexual health quality improvement program
UNSW: University of New South Wales
UK: United Kingdom
US: United States of America
